# Public Charities

**Published:** 1881-06

**Authors:** 


### Editorial.

### PUBLIC CHARITIES.

The poor as well as the rich become sick and must be cared for. The question arises, who shall assume this work? Are those of any particular profession or avocation under special obligations? No one, having the means, we answer, is exempt, and the merchant and farmer have no more right to refuse food and raiment than has the doctor to decline a call for his services and medicine. The famishing invalid can more certainly lengthen out the thread of life without medicine than bread; so that the producer of the staff of life often has upon him more responsibility for the life of a human being than the so-called guardian of health and life. The rich planter, merchant or lawyer rants and fumes if the weary doctor refuses to ride five miles to see a case of intermittent fever in the family of a pauper, and at the same time objects to a proposition looking to the expenditure of one dollar for his sustenance. The idea is promulgated, and by silly peop'e entertained, that the doctor is sworn to render medical aid to afflicted paupers, and even those who know better contend that the physician's services should be rendered gratuitously, since they cost him nothing. Such persons forget that his profession is his stock in trade, as well as that of the merchant, and that his medicine, which the country doctor is expected to furnish, has cost him the cash, earned by midnight toil and care for the more fortunate (financially) of his patients. Doctors are human beings, requiring means for the support of themselves and families, like other people, and if their time and scanty means are devoted to those not able to remunerate them, their career as practicing physicians must speedily close.

While this is true, we claim that ours is really a humane profession, and that no class of citizens does more in the cause of humanity than medical men. Of this we are proud, for it is in the line of morality and the Christian religion, but there is no common sense in killing the goose to get the golden egg.

There is, however, a plan adopted by all well regulated cities, and many counties, by which the burden of this munificence is pretty equally divided among well to do people. Hospitals are organized and supported at public expense, in which indigent invalids are made comfortable and the proper means afforded for their restoration to health. Under this arrangement the medical man does more than others, for, while he pays the same taxes as others, his professional services are often afforded gratuitously. This, however, is a great improvement upon that system which requires the immense labor of visiting them at their several homes and furnishing medicine at his own expense.

The abominable plan pursued by this and some other Southern cities, of electing a certain number of physicians to attend the sick poor, at a price but little more than is necessary to buy the required amount of medicine, relieves the profession generally from the supposed obligation of attending paupers, yet the humane heart is chilled at the sad neglect which must necessarily attend this system The salary affording a mere moiety of support of himself, he is compelled to seek business with paying patients to the neglect of his pauper charge.

The lasting stain of disgrace that marks the city of Atlanta in refusing to found and support a hospital is not likely soon to be wiped out by a change in the policy of the officials. Some praiseworthy efforts have, however, been made by private individuals to lessen the odium heaped upon us, but they have not yet culminated in success. The Ladies' Home and the Sisters' Atlanta Hospital have made efforts in this direction, and each has received a nominal aid from the city, but not sufficient to ensure the accomplishment of the desired object. The medical colleges have also laid plans for this purpose, making at least four distinct propositions for hospitals in the city, each seemingly jealous of the other, and instead of uniting the efforts of all for the great humane purpose, so divide the energy and means that the success of none is probable. A little house with a dozen bunks without means of support, amounts literally to a waste of what has been expended. We now have two or three of this kind, and will probably soon have another. The partizans of each doubtless will appeal to the city authorities for aid, and if they should ever be brought to the rational conclusion that a hospital shou'd be founded and supported by public means they will probably be influenced by political policy to squander a little here and there, to no practical or useful purpose. We wish for better things, but the signs indicate no permanent good from such efforts.

